# Innovative patient-specific delivered-dose prediction for volumetric modulated arc therapy using lightweight Swin-Transformer

**DOI:** 10.3389/fonc.2025.1640685

**Published:** 2025-09-18

**Authors:** Yongqiang Zhou, Changfei Gong, Junming Jian, Yun Zhang

**Affiliations:** ^1^ Department of Radiation and Medical Oncology, First Affiliated Hospital of Wenzhou Medical University, WenZhou Radiation Oncology and Translational Research Key Laboratory, Wenzhou, Zhejiang, China; ^2^ Department of Radiation Oncology, Jiangxi Cancer Hospital & Institute, Jiangxi Clinical Research Center for Cancer, Nanchang, Jiangxi, China; ^3^ NHC Key Laboratory of Personalized Diagnosis and Treatment of Nasopharyngeal Carcinoma (Jiangxi Cancer Hospital), Nanchang, Jiangxi, China

**Keywords:** deep learning, Swin-Transformer, volumetric modulated arc therapy, pre-treatment specific quality assurance, multimodal

## Abstract

**Background:**

Volumetric modulated arc therapy (VMAT) necessitates rigorous pre-treatment patient-specific quality assurance (PSQA) to ensure dosimetric accuracy, yet conventional manual verification methods encounter time and labor constraints in clinical workflows. While deep learning (DL) models have advanced PSQA by automating metrics prediction, existing approaches relying on convolutional neural networks struggle to reconcile local feature extraction with global contextual awareness. This study aims to develop a novel lightweight DL framework that synergizes hierarchical spatial feature learning and computational efficiency to enhance VMAT-delivered dose (VTDose) prediction.

**Methods:**

We propose a hybrid architecture featuring a novel hierarchical fusion framework that synergizes shifted-window self-attention with adaptive local-global feature interaction. (termed “STQA”). Specially, strategic replacement of Swin-Transformer blocks with ResNet residual modules in deep layers, coupled with depthwise separable attention mechanisms, enables 40% parameter reduction while preserving spatial resolution. The model was trained on multimodal inputs and evaluated against state-of-the-art methods using structural similarity index (SSIM), mean absolute error (MAE), root mean square error (RMSE), and gamma passing rate (GPR).

**Results:**

Visual evaluation of VTDose and discrepancy maps across axial, coronal, and sagittal planes demonstrated enhanced fidelity of STQA to ground truth (GT). Quantitative analysis revealed superior performance of STQA across all evaluation metrics: SSIM=0.978, MAE=0.163, and RMSE= 0.416. GPR analysis confirmed clinical applicability, with STQA achieving 95.43%±3.41% agreement with GT (94.63%±2.84%).

**Conclusions:**

STQA establishes a paradigm for efficient and accurate VTDose prediction. Its lightweight design, validated through multi-site clinical data, addresses critical limitations in current DL-based PSQA, offering a clinically viable solution to enhance radiotherapy PSQA workflows.

## 1 Introduction

Volumetric modulated arc therapy (VMAT) has emerged as a cornerstone of precision radiotherapy, achieving superior dose conformity through synchronized dynamic multi-leaf collimator (MLC) modulation and gantry rotation (1). While this technological complexity enhances treatment plan quality compared to conventional techniques, it simultaneously intensifies the demand for rigorous verification of dose distribution authenticity and deliverability. Pre-treatment patient-specific quality assurance (PSQA) remains an essential clinical safeguard, strongly endorsed by the American Association of Physicists in Medicine (AAPM) to ensure VMAT dose accuracy and patient safety (2). Current clinical workflows employ measurement devices such as diode arrays, ionization chambers, and radiographic films to quantify discrepancies between planned and delivered dose. However, conventional PSQA workflows, which depend on physical measurements, are time-consuming and labor-intensive. They delay treatment initiation and reduce the efficiency of radiotherapy services (3).

Over the past decade, machine learning (ML) has driven advancements in PSQA, particularly in gamma passing rate (GPR) prediction. Early ML approaches, including Poisson regression with Lasso regularization for binary classification (4, 5), regression/classification models for VMAT plans (6), artificial neural networks (ANN) for dosimetry prediction (7), and feature-engineered support vector machines (8, 9), demonstrated moderate success but faced limitations in accuracy and clinical applicability due to manual feature dependency. The emergence of deep learning (DL) revolutionized this field through automated hierarchical feature extraction via convolutional neural networks (CNN). Key innovations include CNN architectures for prostate cancer PSQA (10, 11), transfer learning-enhanced VGG-16 models outperforming domain-expert systems (12), fluence map-based error detection frameworks (13), GANs for EPID-to-dose conversion (14), and log file-informed fluence modeling (15–18). By eliminating manual feature engineering and enabling end-to-end prediction through raw data abstraction, DL methods have significantly improved prediction accuracy and clinical utility compared to traditional ML approaches, establishing a paradigm shift in PSQA optimization.

Extensive studies have validated the potential of ML/DL models in terms of predicting PSQA without performing real measurements (4–18). However, critical analysis of existing methodologies reveals three fundamental limitations requiring attention for clinical implementation of ML/DL-based PSQA models. Firstly, the predominant GPR evaluation paradigm fails to establish quantitative relationships between spatial dose distribution characteristics and validation outcomes, particularly at anatomically complex sites. This limitation obscures detection of subclinical dose deviations and provides insufficient spatial context (e.g., failure point localization, clustered anomalies) for comprehensive clinical assessment (19, 20). Secondly, most models rely on 2D planar dose representations, inherently incapable of capturing the 3D spatial modulation characteristics intrinsic to VMAT’s dynamic delivery. This dimensional reduction introduces systematic errors in dose carving pattern recognition. Thirdly, while CNN excel at local feature extraction, their reliance on downsampling operations sacrifices spatial resolution and local detail preservation. The inherent locality of convolutional kernels further restricts global contextual awareness and long-range spatial relationships modeling - critical capabilities for holistic dose distribution analysis.

The remarkable success of Transformers in natural language processing (21) has spurred their adaptation to computer vision, leveraging global self-attention mechanisms to overcome the local inductive bias inherent in CNNs. Pioneering this shift, Kolesnikov et al. developed the Vision Transformer (ViT) (22), achieving state-of-the-art image recognition through patch-based sequence processing. Recent work by Zeng et al. (23) demonstrates a hybrid network integrating Transformers with modified U-Net architectures for predicting measurement-guided volumetric dose in PSQA, enabling quantitative analysis of spatial dose differences between predicted and clinical dose distributions. However, subsequent studies reveal critical limitations of pure Transformer architectures in vision tasks, particularly their inadequate local feature extraction capabilities for dense predictions (24–27). This limitation has motivated hybrid architectures combining CNN and Transformer encoders through serial (e.g., TransUNet (28)) or parallel (e.g., TransFuse (29)) configurations to synergize global context modeling with local feature learning. Concurrently, enhanced variants like Swin Transformer (30) incorporate hierarchical shifted-window mechanisms, demonstrating superior performance in pixel-level prediction tasks and advancing the evolution of vision-specific Transformer architectures.

To address the critical limitations in existing PSQA methodologies, we propose STQA (Swin Transformer-based Quality Assurance) - a novel lightweight network that synergizes hierarchical feature learning with adaptive global-local attention for volumetric dose prediction in VMAT-PSQA. Departing from conventional Transformer adaptations, our architecture introduces three key innovations: 1) A depth-aware hierarchical encoder-decoder framework employing parameter-shared shifted window attention across scales, enabling efficient cross-resolution feature interaction while preserving spatial fidelity; 2) A dual-path feature extraction mechanism combining depth-wise separable local attention with global context modeling through lightweight transformer blocks, effectively capturing both fine-grained dose carving patterns and long-range anatomical dependencies; 3) Bottleneck-adapted skip connections with channel-wise excitation modules that dynamically recalibrate multi-scale features during spatial resolution recovery. Extensive experiments demonstrate STQA’s capability to predict 3D dose distributions closely matching actual VTDose, enabling patient-specific VTDose acquisition. Our method not only demonstrates superior overall prediction performance but also consistently outperforms comparative models across multiple cancer sites (head & neck, chest, abdomen). Significantly, STQA achieves a 40% parameter reduction versus Swin Transformer through depth-wise separable attention in shallow layers, hierarchical parameter-shared window processing, and bottleneck adapters within skip connections that strategically compress and reactivate channels, thereby maintaining performance while eliminating architectural redundancy.

## 2 Methods

### 2.1 Data collection and preprocessing

The study cohort comprised 200 patients treated with volumetric modulated arc therapy (VMAT) between 2020 and 2024 (**Table 1**) in Jiangxi Cancer Hospital. The original dataset is split into training (160), validation (20), and test set (20), which contain 7731, 1045 and 1105 images, respectively. All computed tomography (CT) simulations were performed using a Somatom Confidence RT Pro CT scanner (Philips Healthcare, Best, the Netherlands) with 2 mm slice thickness. To ensure precise target delineation, coregistered diagnostic magnetic resonance imaging (MRI) and positron emission tomography (PET) images were integrated into the planning process by board-certified radiation oncologists with >10 years’ experience in radiotherapy. VMAT plans were generated using clinically validated treatment planning systems: the Monte Carlo algorithm in Monaco (version 5.11, Elekta AB) with a dose calculation grid of 2 mm. All plans were optimized through multi-criteria iterative optimization to ensure optimal target coverage while adhering to strict organ-at-risk dose constraints. Finalized plans were delivered via 6 MV flattening filter-free beams using an Elekta Infinity linear accelerator equipped with a 160-leaf Agility multileaf collimator (MLC). Prior to treatment, comprehensive quality assurance was performed using the ArcCHECK-3DVH system (Sun Nuclear Corporation, Melbourne, FL, USA), which underwent comprehensive calibration procedures including validation array measurements, beam modeling verification (gamma pass rate >95% at 3%/3 mm), and dose reconstruction accuracy assessments.

**Table 1:** Clinical characteristics of cancer patients enrolled in this study.

Characteristics	Sample number	Percentage
Gender, no. (%)		
Male	120	60.0%
Female	80	40.0%
Age (years)		
<20y	15	7.5%
20y-60y	100	50.0%
>60y	85	42.5%
Cancer sites		
H&N	38	19.0%
Chest	116	58.0%
Abdomen	46	23.0%

To ensure spatial consistency across all data types, both the measured and TPS-planned dose distributions were extracted directly from DICOM RT Dose files and converted into 32-bit floating-point arrays (3). These dose maps were then interpolated to align with the coordinate system of the corresponding CT images and resampled to a uniform grid resolution. Each 3D volume—including CT, planned dose, and measured dose—was initially represented as a matrix of size 512 × 512 × 150 pixels. Zero-padding was applied during interpolation to preserve spatial dimensions. To optimize computational efficiency and memory usage, all images were down-sampled to a resolution of 256 × 256 × 150 prior to model input. Planned dose values were normalized to the maximum dose value within each plan to facilitate stable network training. The model outputs, which are generated in normalized form, are subsequently denormalized back to absolute dose values in units of Gy by rescaling with the same reference maximum dose. These final predictions are then formatted into DICOM RT-Dose objects compatible with clinical systems, enabling direct use in standard quality assurance procedures such as gamma index analysis and DVH evaluation.

### 2.2 The overall network structure

**Figure 1:**
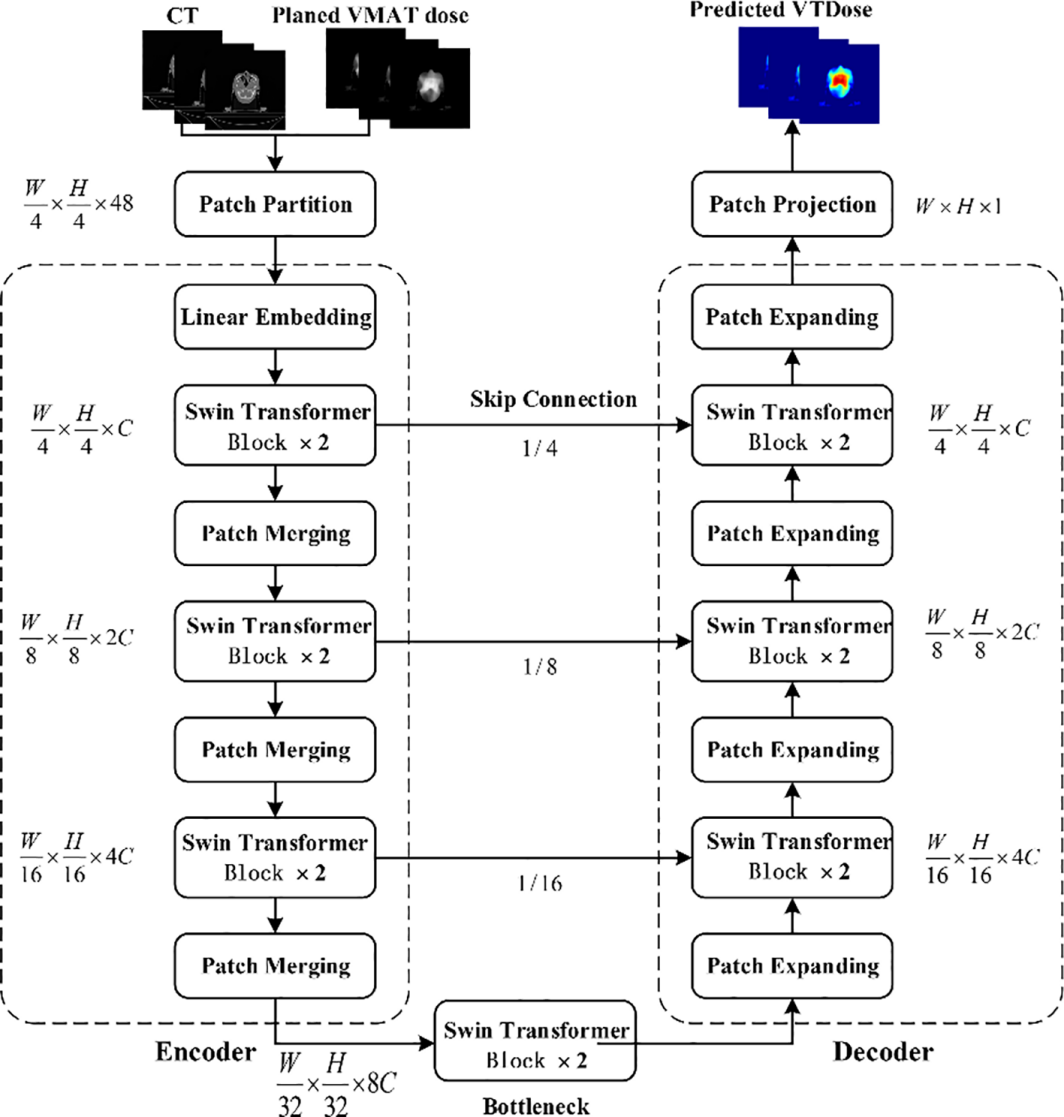
Flow chart of the proposed STQA.

The overall architecture of the STQA network proposed in this study, as illustrated in **Figure 1**, incorporates targeted modifications to the original Swin-UNet framework to better align with our dose prediction objectives. To address the specific requirements of our task and enhance computational efficiency, we implemented two key architectural adjustments: first, replacing consecutive Swin Transformer blocks at the bottleneck layer with final residual network components of ResNet to capitalize on the inherent advantage of residual blocks in maintaining feature extraction capacity while mitigating computational complexity, while preserving original image resolutions and feature dimensions; second, strategically substituting both the loss function and optimization algorithm to facilitate stable training convergence and improve task-specific adaptation. Crucially, STQA retains the essential U-shaped configuration comprising four core components - encoder, bottleneck, decoder, and skip connections - as visually demonstrated in **Figure 1**, ensuring effective feature propagation and multi-scale information integration throughout the network architecture.

### 2.3 Swin-Transformer-based feature extraction

**Figure 2:**
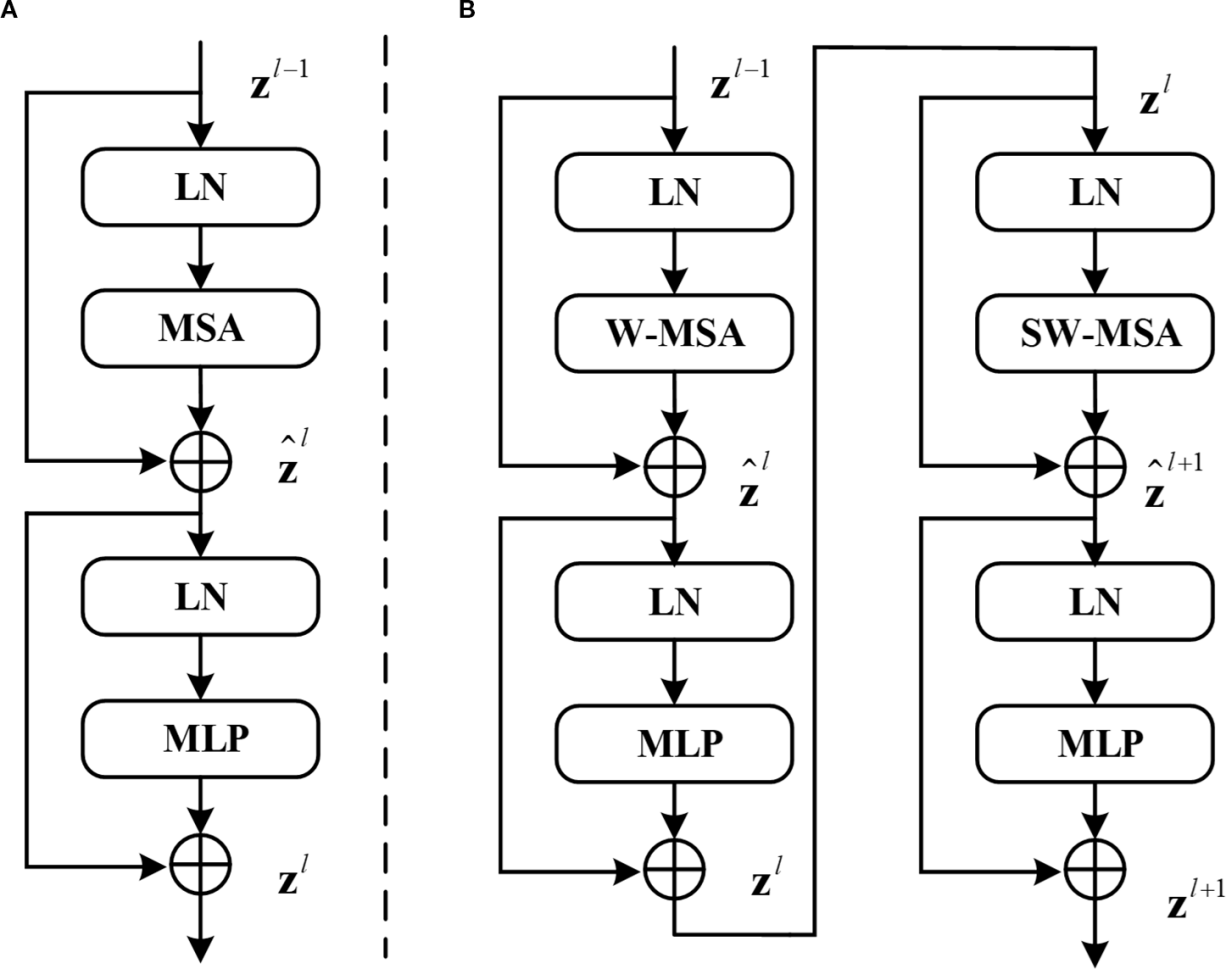
**(A)** Structure of the standard transformer block. **(B)** Two consecutive Swin transformer blocks (renamed W-Trans block and SW-Trans block).

The Swin Transformer architecture employs two distinct attention mechanisms as its core feature extraction components: the Window Multihead Self-Attention (W-MSA) module that processes localized image regions through fixed window partitioning, and the Shifted Window Multihead Self-Attention (SW-MSA) module that enables cross-window information exchange through strategic window shifting operations, with their hierarchical arrangement and interaction patterns visually detailed in **Figure 2**.

SwinUNet utilizes Swin-Transformer layers for feature extraction, Patch Merging and Patch Expanding layers for downsampling and upsampling respectively and incorporates skip connections inspired by U-Net to fuse encoder features in the decoder.


(1) $${\hat{z}}^{l}=\text{W}-\text{MSA}(\text{LN}(z^{l-1}))+z^{l-1}$$

(2) $$z^{l}=\text{MLP}(\text{LN}({\hat{z}}^{l}))+{\hat{z}}^{l}$$

(3) $${\hat{z}}^{l+1}=\text{SW}-\text{MSA}(\text{LN}(z^{l}))+z^{l}$$

(4) $$z^{l+1}=\text{MLP}(\text{LN}({\hat{z}}^{l+1}))+{\hat{z}}^{l+1}$$

(5) $$Attention(Q,K,V)=SoftMax(\frac{QK^{T}}{\sqrt{d}}+B)V$$

In Equations 1–4, ${\hat{z}}^{l}$ and $z^{l}$ denote the outputs of the l−th ‘s (S)W-MSA model and the MLP model respectively. In Equation 5, $Q,K,V\in R^{M^{2}\times d}$ represent the query matrix, key matrix, and value matrix respectively. $M^{2}$ represents the number of patches in a window, while d denotes the dimension information of the query or key matrix. Due to the fact that the axis values of relative positions in the model are all within[-*M*+1,*M*+1], a smaller deviation matrix needs to be parameterized as $\hat{B}\in R^{\left(2M-1)\times (2M+1\right)}$ , where B is the value fetched from $\hat{B}$ . In Swin Transformer blocks, the input data first pass through a LayerNorm (LN) layer. LN here serves a similar role to BatchNorm (BN) commonly used in Computer Vision (CV). Both are designed to normalize the activations of the previous layer to some extent to avoid the vanishing gradient problem. The difference between LN and BN lies in the dimensions over which normalization is computed. LN computes normalization across the layer dimension, whereas BN computes it across the batch dimension. In the field of NLP, the batch size of networks is typically smaller than in CV, making BN less effective compared to LN. Therefore, LN layers are commonly used in Transformers. The formula for LN is shown in Equation 6.


(6) $$y=\frac{x-\text{E}[x]}{\sqrt{\text{Var}[x]+\epsilon }}*\gamma +\beta$$

Where E[x] represents the mean of x and Var[x] represents the variance of x . ϵ is a very small number to avoid the possibility of zero denominator, γ and β are learnable parameters.

After passing through the LN layer, it is input into the W-MSA or SW-MSA layer. Compared to multi-head self-attention (MSA), W-MSA saves a significant amount of computation by independently computing each window. For an input image of size (h,w) , assuming each window contains patches of size M×M, the computational complexity formulas for MSA and W-MSA are given by Equations 7, 8 respectively.


(7) $$\Omega (\text{MSA})=4hwC^{2}+2{\left(hw\right)}^{2}C$$

(8) $$\Omega (\text{W}-MSA)=4hwC^{2}+2M^{2}hwC$$

W-MSA reduces computation but leads to a lack of information communication between windows. To address this issue, SW-MSA must be computed in subsequent blocks. Information interaction between windows is achieved by shifting the windows down and to the right by half the window size and then computing W-MSA again for the shifted windows. Therefore, W-MSA and SW-MSA need to appear in pairs. It is for this reason that the number of blocks in Swin Transformer is typically even. In Swin-UNet, the number of blocks in Swin Transformer is 2, comprising one W-MSA block and one SW-MSA block. After passing through the W-MSA layer or SW-MSA layer, followed by a BN layer, and finally a multi-layer perceptron (MLP) for feature mapping, the final output is obtained.

### 2.4 The proposed STQA

**Figure 3:**
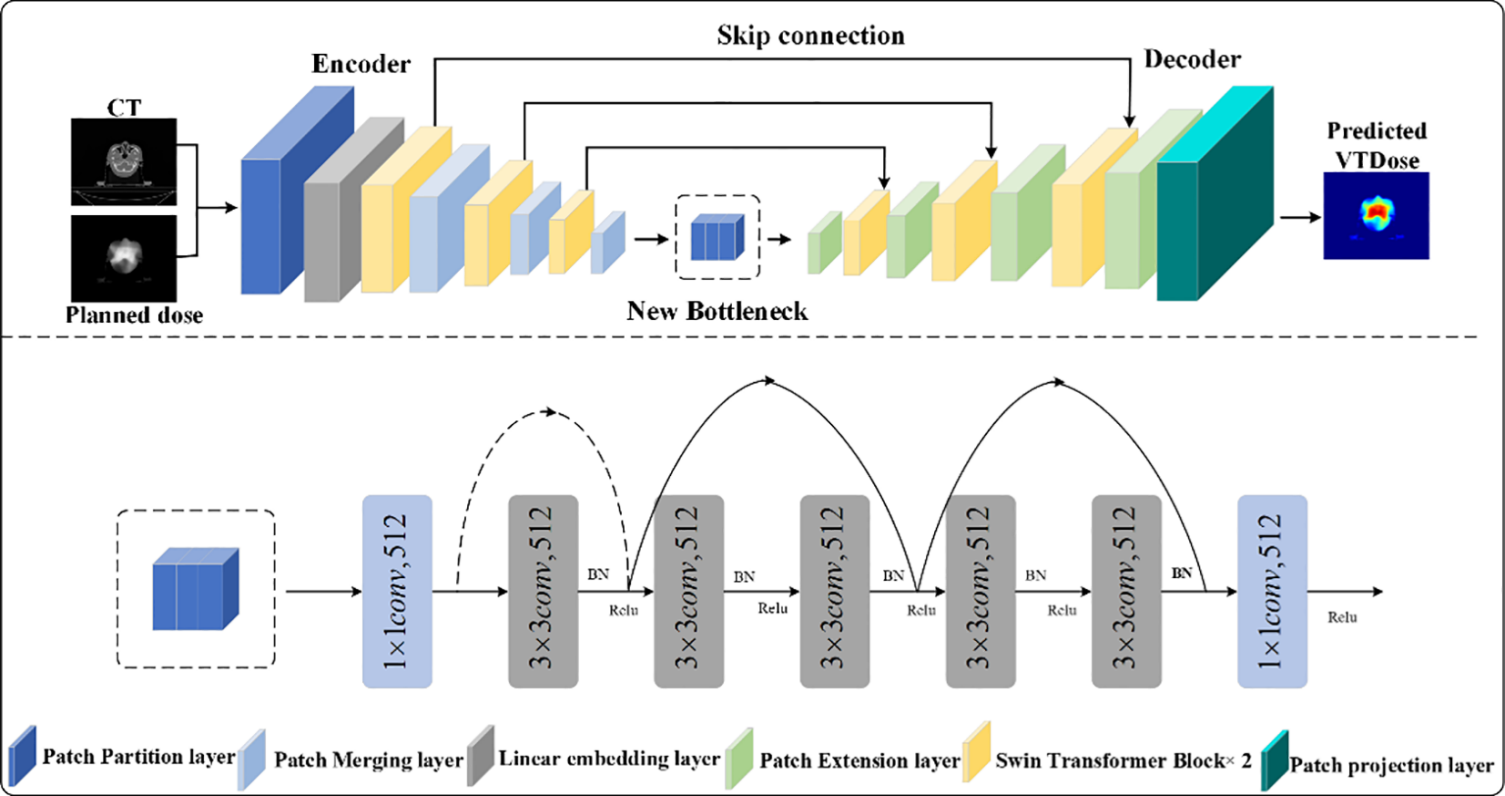
The proposed STQA network.

Swin-UNet demonstrates powerful capabilities in extracting contextual information and restoring spatial resolution; however, the convergence of transformer modules for image feature computation in deep bottleneck sections remains suboptimal. Considering the challenges of network parameterization as depth increases, this paper proposes enhancements to the deep bottleneck of Swin-UNet. Since the design of residual blocks in ResNet does not reduce feature extraction capacity with increased network depth, replacing two consecutive Swin Transformer blocks in the bottleneck position with ResNet layers is a viable solution. ResNet networks, primarily composed of multiple residual modules—a popular structure in modern neural networks—address the degradation issues caused by deepening layers, thus enabling parameter computation even in thousand-layer networks. After optimization and comparison, we adopt the final layer of the deep ResNet network as the bottleneck of Swin-UNet to improve the model’s predictive accuracy in quality assurance of preprocessing patient-specific data, as illustrated in **Figure 3**; additionally, to reduce parameter computation, 1×1 convolutions are employed for dimensionality reduction on feature vectors.

As data features pass through the last layer of the ResNet deep network, both image resolution and feature dimensions remain unchanged. As shown in **Figure 3**, this layer comprises three residual blocks, with each residual module consisting of a residual block layer that includes two convolutional blocks, two BN layers, and one ReLU activation. The improved Swin-UNet network maintains the same encoder, bottleneck, and decoder components as the original, but it replaces the Swin-UNet bottleneck with the last layer of the ResNet network—resulting in nearly a 40% reduction in network parameters while achieving better performance.

In the encoder, the image is first divided into patches using a Patch Partition layer, and a linear embedding layer tokenizes the data to produce a C-dimensional representation of size H/4 × W/4. The divided blocks are then concatenated via a Patch Merging layer, which reduces the patch resolution to half of the original; although the merged features are initially four times the original dimension, an additional linear layer is applied to unify the dimension to twice the original. At the bottleneck, leveraging the advantage of ResNet’s residual blocks that do not degrade in performance as the network deepens, the fifth layer structure of ResNet is employed to overcome the convergence issues of transformer blocks in deep networks, with the input feature resolution set at W/32 × H/16 and remaining unchanged. Finally, the Patch Expanding layer upsamples the features by doubling the resolution while halving the feature dimension until full-size resolution is restored, and the skip connections fuse multi-scale features from the encoder with the upsampled features to mitigate spatial information loss caused by downsampling. The algorithm flow of STQA is as follows: (see **Algorithm 1**)

Algorithm 1 **STQA.**

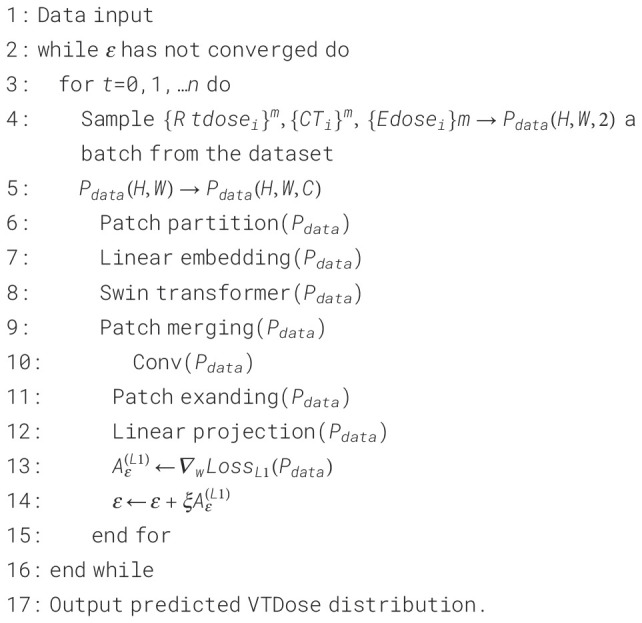

### 2.5 Experiment setup

To validate the effectiveness of STQA predictions, we compared our method with three established prediction networks using the same test set: U-Net (31), CycleGAN (CGAN) (30), TransQA (TrQA) (23), and Swin-UNet (SWNet) (30). The compared methodologies are summarized as follows: (1) U-Net: A classical encoder-decoder architecture recently adapted for dose prediction tasks (31), demonstrating strong performance in medical image analysis. (2) CGAN: An unsupervised framework proposed by Zhu et al. (32) that employs dual generative adversarial networks with cycle consistency, eliminating the requirement for paired training data. (3) TrQA: A hybrid architecture integrating Transformer’s self-attention mechanisms with enhanced U-Net structures, specifically designed for VTDose prediction in PSQA (23). (4) SWNet: A pioneering U-shaped network developed by Lin et al. (30) that incorporates hierarchical Swin Transformer blocks in both encoder and decoder pathways to improve medical image segmentation.

For quantitative evaluation, we adopted three established metrics: structural similarity index (SSIM), mean absolute error (MAE), and root mean square error (RMSE). The experimental dataset comprised paired radiotherapy planning data including CT images, Planned dose distributions, and corresponding VTDose ground truth (GT) maps, collected from multiple cancer patients. To leverage multimodal information, we concatenated CT and Planned dose images along the channel dimension as dual-channel inputs, preserving their distinct information characteristics while providing complementary anatomical and dosimetric features to the network. In addition, GPR analysis serves as the most widely adopted methodology for comparing measured and calculated dose distributions in PSQA for VMAT, where the agreement level is typically quantified through GPR metrics. To further evaluate the prediction accuracy across different methods, we additionally compared the three-dimensional GPR (3%/2mm criterion with a 10% threshold) of various prediction approaches.

The proposed STQA architecture was implemented in PyTorch and trained/tested on an NVIDIA GeForce RTX 3090 GPU with 16GB memory using CUDA-accelerated computation. We employed the Adam optimizer with L1 loss as the primary objective function, setting the initial learning rate to 1e-5 and training for 200 epochs. To ensure fair comparison, all baseline models were re-implemented using identical training protocols and hardware configurations. The total training time for each model was recorded as follows: U-Net: 28 hours, CGAN: 34 hours, TrQA: 41 hours, SWNet: 44 hours, and STQA: 38 hours. After training, each model can generate a full 3D dose distribution within approximately 5–7 seconds, demonstrating compelling inference speed suitable for time-sensitive clinical settings.

Ablation studies were conducted to systematically evaluate key architectural components and parameter settings in our framework. The investigation comprised two main aspects: (1) Performance comparison among three architectural variants: baseline Swin-UNet, our full STQA model, and a hybrid Swin-UNet+ResNet (SURNet) configuration with ResNet blocks directly cascaded at the bottleneck layer. (2) Quantitative analysis of skip connection configurations in STQA, where different numbers of cross-scale connections (0-3) were tested. Specifically, 3 skip connections represent full connections at 1/16, 1/8, and 1/4 resolution levels; 2 connections utilize 1/16 and 1/8 levels; 1 connection employs only the 1/16 level, while 0 connections indicate complete removal of skip connections. This systematic evaluation enables comprehensive understanding of feature propagation mechanisms in our proposed architecture.

## 3 Results

**Table 2** presents the quantitative evaluation results across all test cases. As demonstrated in **Table 2**, STQA achieves statistically significant improvements over U-Net and CGAN across all metrics. When comparing STQA with the state-of-the-art methods TrQA and SWNet, our method exhibits superior performance, particularly in the RMSE metric, where STQA reduces the error to 0.416 compared to 0.646 for TrQA and 0.597 for SWNet. In terms of structural similarity, STQA achieves an SSIM value of 0.978, outperforming TrQA (0.958) and SWNet (0.944) by margins of 0.034 and 0.020, respectively.

**Table 2:** Comparison of experiments based on STQA and other prediction network models.

Method	SSIM	MAE(%)	RMSE(%)
U-Net	0.788	0.608	0.931
CGAN	0.891	0.419	0.867
TrQA	0.944	0.251	0.646
SWNet	0.958	0.198	0.597
STQA	0.978	0.163	0.416

**Figure 4:**
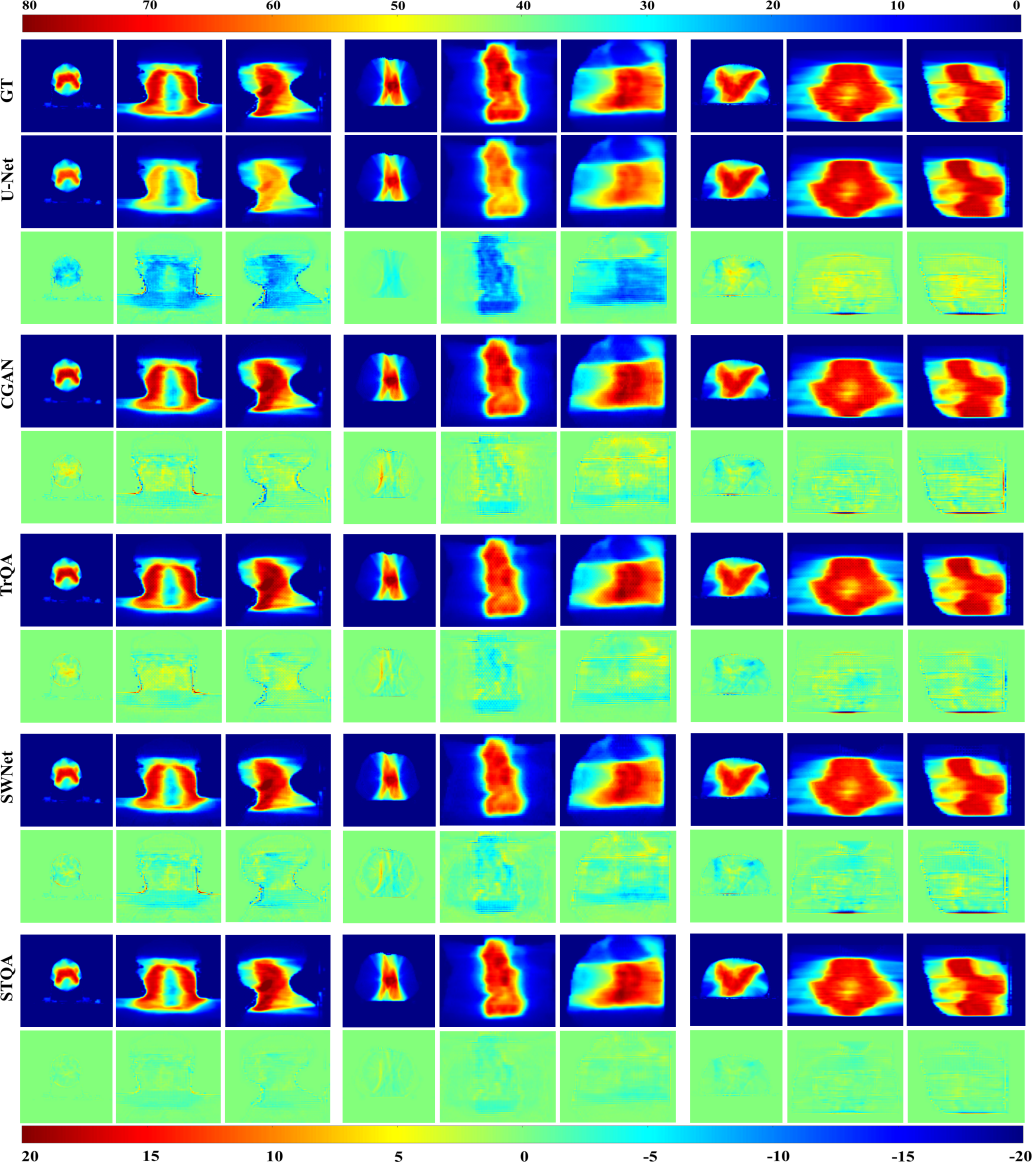
Qualitative analysis of predicted VTDose distributions (in Gy) across methodologies. Dose distributions are visualized for head & neck (columns 1-3), chest (columns 4-6), and abdominal (columns 7-9) cases. Rows 3, 5, 7, 9, and 11 demonstrate dose discrepancy maps between GT and predicted results. Anatomical plane assignments follow: columns 1/4/7 display axial dose distributions, columns 2/5/8 depict coronal plane mappings, and columns 3/6/9 correspond to sagittal plane patterns.

For enhanced visual comparison across methodologies, **Figure 4** presents representative predicted dose distributions spanning three anatomical regions (head & neck, chest, abdomen) in axial, coronal, and sagittal orientations. Visual inspection of **Figure 4** demonstrates that U-Net and CGAN underperform relative to the comparative methodologies, with U-Net exhibiting the most pronounced prediction inaccuracies. The VTDose maps indicate that STQA generates predictions with enhanced dose fidelity, a finding further supported by comprehensive analysis of dose difference maps. Comparative evaluation of discrepancy distributions reveals that Transformer-based models (TrQA, SWNet, and STQA) exhibit significantly reduced deviations compared to conventional approaches. Notably, STQA achieves minimal dose discrepancies across all clinical cases, outperforming other Transformer-based counterparts in maintaining alignment with GT dose distributions. To assess local dose accuracy, we computed mean absolute errors for Dmean and Dmax in critical OARs including the spinal cord and parotid glands. STQA achieved errors of 1.08 ± 1.21 Gy and 1.14 ± 0.67 Gy, respectively, outperforming all baselines, followed by SWNet and TrQA, UNet has the worst. This performance advantage suggests STQA’s superior capability in preserving dosimetric details while ensuring spatial consistency with GT.

To evaluate the predictive performance of each network for specific cancer sites, tests were conducted separately based on three major cancer sites (head & neck, chest, abdomen), and the results of each method were compared as shown in **Table 3**. From the comparison across the three metrics, all methods exhibited better dose prediction results for the chest than the other two sites. This may be due to the simpler structure of the thorax compared to the other two sites and the fact that chest patients accounted for the largest number (116, 58%), making it easier for the network to extract features. Additionally, the prediction accuracy of abdominal patients is slightly better than that of head and neck patients, which is likely due to the small number of head and neck patients and the complex anatomical structure. Despite the imbalanced distribution of cancer sites, stratified sampling during data splitting helped mitigate bias, and STQA consistently outperformed baselines across all sites. Overall, STQA achieved the best predictive accuracy across all three cancer sites. This indicates that the STQA network demonstrates the best performance across various shapes and texture differences. Furthermore, the GPR analysis revealed distinct performance differences among models: The U-Net model achieved suboptimal GPR results (98.54 ± 3.42%), showing statistically inferior performance compared to other methods. In contrast, STQA demonstrated the closest agreement with GT measurements, yielding GPR values of 95.43 ± 3.41% versus the GT baseline of 94.63 ± 2.84%. Intermediate performance was observed for CGAN (98.22 ± 2.74%), TrQA (96.91 ± 4.16%), and SWNet (96.20 ± 3.65%), all showing comparable GPR outcomes. The mean errors between the GPR of the VTDose and the predictions were 4.24% for the U-Net, and 3.42%, 2.52%, 1.77%, 1.1% for CGAN, TrQA and STQA, respectively.

**Table 3:** Comparison of model performance across different cancer sites.

Method	SSIM	MAE(%)	RMSE(%)
	H&n/abdomen/chest	H&n/abdomen/chest	H&n/abdomen/chest
U-Net	0.782/0.816/0.821	0.522/0.513/0.505	0.865/0.841/0.822
CGAN	0.892/0.898/0.901	0.419/0.400/0.381	0.848/0.826/0.805
TrQA	0.948/0.954/0.966	0.250/0.245/0.225	0.637/0.632/0.5724
SWNet	0.964/0.967/0.971	0.195/0.186/0.162	0.583/0.577/0.468
STQA	0.980/0.984/0.985	0.159/0.152/0.145	0.411/0.408/0.365

**Table 4** illustrates the ablation experiment of the performance differences among different model architectures. In the comparison of parameter quantities among the three structural models, we observed that replacing the bottleneck of the original Swin-UNet with ResNet’s network layers (STQA) resulted in a reduction of nearly 40% in the memory footprint of the trained model files. Additionally, STQA exhibited a reduction of almost 50% in model file memory compared to SWNet. This indicates that the STQA architecture not only reduces redundant parameters and has a smaller time complexity but also slightly improves performance. While the SURNet model exhibits the best performance, its deeper network structure leads to larger model parameter quantities and higher time complexity. Therefore, considering all factors, we believe that the STQA structure demonstrates the optimal performance. **Table 5** demonstrates the impact of the number of skip connections in the network on its performance (ablation experiment 2). We observed that the neural network exhibited the highest predictive accuracy when having 3 skip connections. This is likely because an appropriate number of skip connections can effectively integrate features from different layers, enhancing the network’s ability to capture multi-scale information. Too few skip connections may not fully utilize the feature hierarchy, while too many could introduce unnecessary complexity and potential overfitting. Therefore, in this study, we default the number of skip connections to be 3, as it strikes a balance between feature integration and model complexity, leading to optimal performance.

**Table 4:** Comparison of performance and parameters among different model architectures.

Method	SSIM	MAE(%)	RMSE(%)	Model_size
SWNet	0.951±0.5e-3	0.188±0.05	0.587±0.24	98.1MB
STQA	0.982±0.5e-3	0.160±0.04	0.418±0.31	45.2MB
SURNet	0.988±0.4e-3	0.155±0.02	0.394±0.14	105.4MB

**Table 5:** Impact of the number of skip connections on network performance.

Skip connection	SSIM	MAE(%)	RMSE(%)
0	0.815	3.256	8.032
1	0.641	1.577	3.412
2	0.957	0.193	0.543
3	0.977	0.168	0.444

## 4 Discussion

Artificial intelligence, particularly deep learning (DL) techniques, has found extensive application in multiple facets of radiotherapy treatment planning and delivery, such as tumor target delineation (33), adaptive radiotherapy plans (34), 3D dose prediction (23), and PSQA (35). Accurate and rapid implementation of quality assurance processes for patients’ radiotherapy treatments can assist physicists in patient care. In terms of methods, compared to CNN networks, DL networks based on Transformers lack some important inductive biases (e.g., locality and translation equivariance), making their training heavily reliant on large-scale datasets and pre-trained models. However, due to the lack of large-scale and well-annotated datasets, the development of DL in the field of medical imaging lags behind that of natural image processing. In particular, there have been few studies applying Transformers to the field of radiotherapy quality assurance. Recently, Hu et al., proposed a U-shaped network called TrDosePred (36), which consists of convolutional patch embedding and several Transformer blocks based on local self-attention. This network aims to generate dose distributions from contour CT images. The dose score on the test dataset was 2.426 Gy, and the DVH score was 1.592 Gy. The results demonstrate that the performance of TrDosePred is comparable to or even better than previous state-of-the-art methods, proving the potential of Transformers in improving treatment planning processes.

In this paper, we aim to obtain global contextual information from radiotherapy volume images to improve the accuracy of VMAT quality assurance. We innovatively improved the Swin-UNet architecture to construct the STQA network, making the network suitable for handling radiotherapy planning data. Specifically, we modified the loss function and optimizer for training the network to L1 loss and Adam, respectively. Moreover, to explore optimal network training, we attempted to train the network using a combination of two loss functions, L1 and L2, with weighted allocation. Most importantly, we replaced two consecutive Swin Transformer modules between the downsampling and upsampling layers of the Swin-UNet network with ResNet layers to overcome the problem of feature extraction degradation due to network depth, thereby improving performance. The inherent properties of Transformers allow them to handle feature representations at a stable and relatively high resolution, accurately meeting the demands for finer-grained and globally consistent predictions in dense prediction tasks. Compared to other state of the art models, we applied Transformer-based DL methods to the VTDose prediction task and achieved better accuracy. This further demonstrates the outstanding achievements of Transformers in medical imaging compared to traditional CNN networks, helping to narrow the development gap between medical imaging DL and natural image processing.

Visual comparisons through representative predicted VTDose distributions reinforce these quantitative findings. STQA’s VTDose maps show superior fidelity. The dose difference maps further substantiate this, with STQA exhibiting minimal discrepancies across all cases, especially in high-dose regions and critical anatomical structures. This is particularly important as these areas are often the most challenging to predict accurately due to their complexity and the potential consequences of dosing errors. **Tables 2**–**4** demonstrate that our proposed STQA framework achieves state-of-the-art performance in VTDose prediction across multiple evaluation dimensions. Compared to existing Transformer-based methods (TrQA and SWNet), STQA reduces RMSE by 35.6% and 30.3%, respectively, while improving SSIM by 3.6% and 2.1% over these benchmarks. These advancements almost align with the performance gains reported in recent studies utilizing hybrid architectures for medical image analysis (30). The 16.6-25.3% improvement in SSIM and 18.5–69.5% reduction in MAE for these challenging cases suggests that our multi-scale skip connection strategy and hybrid bottleneck design effectively capture both global contextual relationships and local texture details—a capability not fully realized in pure Transformer architectures (23). The ablation studies further validate STQA’s architectural innovations. The 40–50% reduction in model memory footprint compared to SWNet, while maintaining competitive accuracy, resolves a key practical limitation of Transformer-based models. Although SURNet achieved marginally higher SSIM values (0.988 vs. STQA’s 0.982), its 2.3× greater parameter count and longer inference time render it clinically impractical. Our results thus suggest that STQA successfully balances computational efficiency with prediction accuracy.

Due to the inherent constraints associated with patient data and DL networks, certain discrepancies between predicted and measured results are unavoidable. Addressing these discrepancies in the future involves augmenting the dataset size or refining DL networks through optimization. The patients in the dataset used in this work come from multiple sites, but they are mixed for both training and testing, rather than having one set for training and another for external testing. Since data from different centers may exhibit significant differences, it can affect the effectiveness of training. In the future, balancing data processing or increasing patient data volume will further improve prediction accuracy. However, it is worth noting that while incorporating multi-institutional data could further improve the model’s generalizability by capturing a broader range of anatomical and dosimetric variations, the present study utilized data from a single institution to ensure consistency in imaging and treatment protocols. The inherent rarity and heterogeneity of medical data pose significant challenges to assembling large, diverse multi-center datasets. The predominance of chest cases may introduce a bias toward simpler anatomies, though our model still performed well on more complex sites. Future work will aim to collect a more balanced dataset across cancer sites and institutions. While we did not separately compute voxel-level sensitivity/specificity for gamma-fail classification, operating directly on volumetric VTDose provides the spatial observability required for fail-voxel localization and *post-hoc* gamma-map synthesis; we plan to report a dedicated voxel-wise gamma-fail analysis in future work. Finally, the model still suffers from time complexity, and we will strive to reduce the model’s time complexity in future work.

In conclusion, this study proposes a new framework termed STQA for VMAT quality assurance, demonstrating superior performance compared to existing models. To strengthen the model’s generalization capacity and convergence properties, we innovatively integrated a ResNet layer into the network’s bottleneck to enhance feature extraction capabilities while adopting advanced loss functions and optimization strategies. Comprehensive validation conducted on VMAT-treated cancer patient datasets revealed that STQA achieves state-of-the-art performance in both global dose distribution prediction and edge dose accuracy across various tumor sites. This successful implementation not only addresses critical challenges in VMAT quality assurance but also paves the way for effective integration of deep learning across medical domains, potentially inspiring novel methodological developments in medical artificial intelligence. From a clinical integration perspective, STQA demonstrates practical feasibility. The average inference time for a full 3D dose prediction is approximately 5–7 seconds on an NVIDIA RTX 3090 GPU, which is compatible with routine QA workflows. The model can be deployed as a standalone application or integrated into existing treatment planning systems via a standardized DICOM RT Dose interface. Future work will focus on user interface development and real-time validation in clinical settings.

## Data Availability

The datasets presented in this article are not readily available because the data used and analyzed during the current study are available from the corresponding author on reasonable request. Requests to access the datasets should be directed to zhangyun_1983@sohu.com.
